# 6-Amino-3,4-dimethyl-4-phenyl-2*H*,4*H*-pyrano[2,3-*c*]pyrazole-5-carbonitrile

**DOI:** 10.1107/S1600536811009354

**Published:** 2011-03-23

**Authors:** Nishith Saurav Topno, Kandhasamy Kumaravel, M. Kannan, Gnanasambandam Vasuki, R. Krishna

**Affiliations:** a Centre for Bioinformatics, Pondicherry University, Puducherry 605 014, India; b Department of Chemistry, Pondicherry University, Puducherry 605 014, India

## Abstract

In the title compound, C_15_H_14_N_4_O, the pyrazole ring is aligned at 88.23 (4)° with respect to the aromatic ring and at 3.75 (4)° with respect to the pyran ring. In the crystal, N—H⋯N hydrogen bonds link adjacent mol­ecules into a linear chain motif. C—H⋯N inter­actions are also observed.

## Related literature

For the synthesis, see: Vasuki & Kumaravel (2008 ▶). For the use of related compounds in organic synthesis, see: Liang *et al.* (2009 ▶). For related structures, see: Kannan *et al.* (2010 ▶).
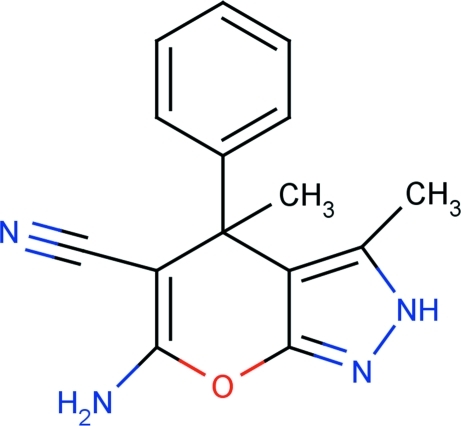


## Experimental

### 

#### Crystal data

C_15_H_14_N_4_O
*M* *_r_* = 266.3
Triclinic, 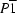

*a* = 6.682 (5) Å
*b* = 9.347 (5) Å
*c* = 11.078 (5) Å
α = 99.213 (5)°
β = 102.740 (5)°
γ = 97.767 (5)°
*V* = 655.6 (7) Å^3^
*Z* = 2
Mo *K*α radiation
μ = 0.09 mm^−1^
*T* = 293 K
0.22 × 0.20 × 0.16 mm

#### Data collection

Bruker APEXII Kappa CCD detector diffractometer
18424 measured reflections
4996 independent reflections
3169 reflections with *I* > 2σ(*I*)
*R*_int_ = 0.036

#### Refinement

*R*[*F* ^2^ > 2σ(*F* ^2^)] = 0.057
*wR*(*F* ^2^) = 0.173
*S* = 0.99
4996 reflections
183 parameters
H-atom parameters constrained
Δρ_max_ = 0.30 e Å^−3^
Δρ_min_ = −0.29 e Å^−3^

### 

Data collection: *APEX2* (Bruker, 2004 ▶); cell refinement: *APEX2* and *SAINT* (Bruker, 2004 ▶); data reduction: *SAINT* and *XPREP* (Bruker, 2004 ▶); program(s) used to solve structure: *SHELXS97* (Sheldrick, 2008 ▶); program(s) used to refine structure: *SHELXL97* (Sheldrick, 2008 ▶); molecular graphics: *ORTEP-3* (Farrugia, 1997 ▶); software used to prepare material for publication: *PLATON* (Spek, 2009 ▶).

## Supplementary Material

Crystal structure: contains datablocks I, global. DOI: 10.1107/S1600536811009354/ng5120sup1.cif

Structure factors: contains datablocks I. DOI: 10.1107/S1600536811009354/ng5120Isup2.hkl

Additional supplementary materials:  crystallographic information; 3D view; checkCIF report

## Figures and Tables

**Table 1:** Hydrogen-bond geometry (Å, °)

*D*—H⋯*A*	*D*—H	H⋯*A*	*D*⋯*A*	*D*—H⋯*A*
N3—H3*A*⋯N1^i^	0.86	2.27	3.129 (2)	173
N3—H3*B*⋯N4^ii^	0.86	2.26	3.087 (2)	160
C10—H10⋯N4^iii^	0.93	2.53	3.455 (3)	172
C14—H14*A*⋯N1^iv^	0.96	2.59	3.522 (3)	163

Symmetry codes: (i) 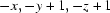; (ii) 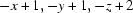; (iii) 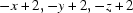; (iv) 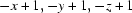.

**Fig. 1.**
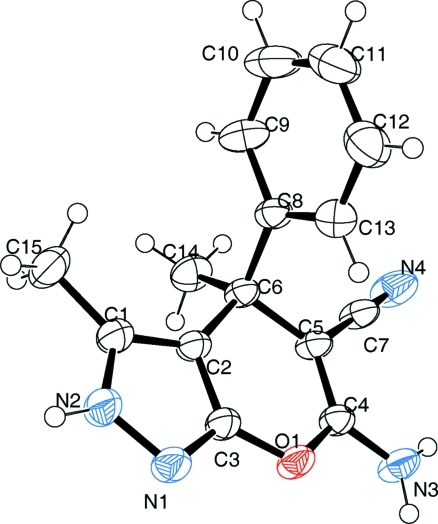
The molecular structure of (I), showing the atom-numbering scheme and displacement ellipsoids drawn at the 50% probability level.

**Fig. 2.**
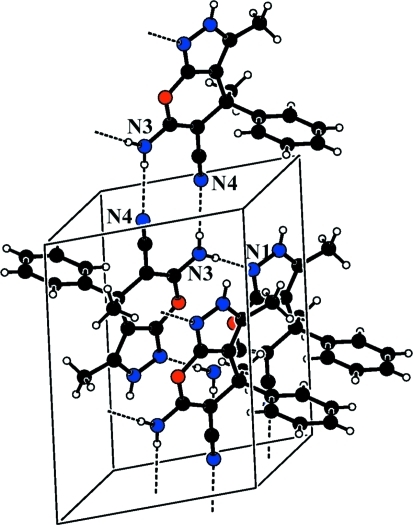
The crystal packing of (I), showing intermolecular hydrogen bonding interactions as dashed lines.

**Fig. 3.**
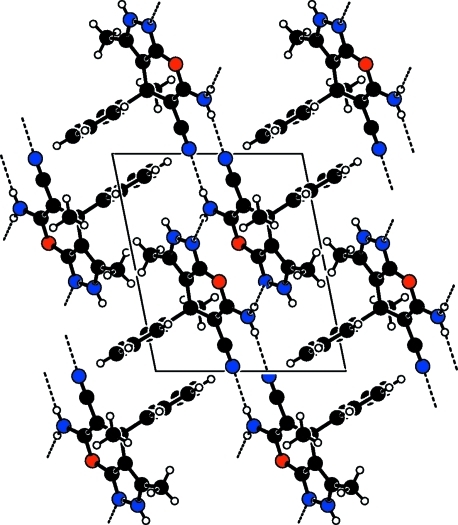
The crystal packing of Compound (I) viewed down the XO-axis, showing intermolecular hydrogen bonding interactions as dashed lines.

**Fig. 4.**
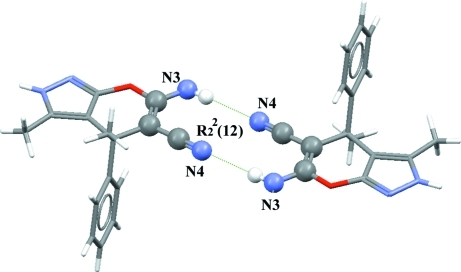
A view of R22 (12) ring motifs formed by N—H···N interaction between two molecules. The ring forming atoms are shown in ball and stick model and the Hydrogen bond are shown in green dashed lines.

**Fig. 5.**
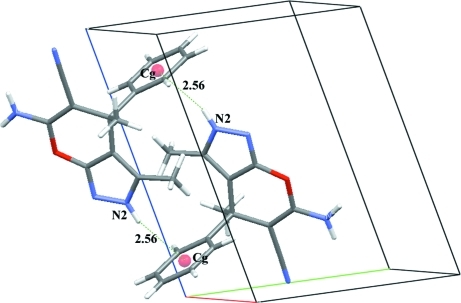
The molecular interaction showing the weak N—H···pi interaction in Compound (I). Cg is a centroid of C8—C13 ring.

## supplementary crystallographic information

### Comment

The pyrano pyrazole derivatives are widely used as organic intermediates (Liang *et al.*, 2009) and are well known for their biological contribution as ChK1 inhibitors. In view of the growing importance of the pyrano pyrazole derivatives, we have synthesized the title compound by Rapid four-component reactions in water (Vasuki & Kumaravel, 2008) and the single-crystal structure analysis was undertaken.

In the title compound, the attached benzyl ring makes the dihedral angle of 88.23 (4) ° and orient in (+)-*syn*-clinal conforamtion with pyrazole ring, whereas the fused pyrazole ring makes 3.75 (4) ° dihedral angle and orient by an (+)-*syn*-periplanar conformation with respect to the pyran ring (Fig. 1). The methyl group is attached to C6 atom of pyran with an angle of 109.29 (11) °. Two N—H···N and C—H···N intermolecular hydrogen bond interactions (Fig. 2) are observed for maintaining the crystal packing (Fig. 3), in which the N3—H···N4 intermolecular interaction are observed to form *R*_2_^2^ (12) ring motifs (Fig. 4). The weak N—H···π intermolecular interaction is also observed for the stabilization of the crystal packing, with a bond distance of 3.382 (3) Å (Fig. 5).

### Experimental

The title compound was prepared by the successive addition of acetophenone 2 (0.240 g, 2 mmol), malononitrile (0.132 g, 2 mmol) and piperidine (5 mol%) to a stirred aqueous mixture of hydrazine hydrate 96% 1 (0.107 g, 2 mmol) and ethyl acetoacetate 2 (0.520 g, 2 mmol) at room temperature under an open atmosphere with vigorous stirring for 5–10 min. The precipitated solid was then filtered, followed by washing with water and then with a mixture of ethyl acetate/hexane (20:80). The product obtained was pure by TLC and 1H NMR spectroscopy. Nevertheless, the products were further purified by recrystallization from ethanol. Analysis calculated for 6-amino-3,4-dimethyl-4-phenyl-2*H*,4*H*-pyrano[2,3-*c*]pyrazole-5-carbonitrile showed that it has C_15_H_14_N_4_O.

### Refinement

All hydrogen atoms were placed in calculated positions, with N—H=0.86 and C—H=0.93–0.97 and included in the final cycles of refinement using a riding model with *U*(H) = 1.2 *U*_eq_(C).

### Crystal data

**Table:**

C_15_H_14_N_4_O	*Z* = 2
*M**_r_* = 266.3	*F*(000) = 280
Triclinic, *P*1	*D*_x_ = 1.349 Mg m^−^^3^
Hall symbol: -P 1	Mo *K*α radiation, λ = 0.71069 Å
*a* = 6.682 (5) Å	Cell parameters from 4350 reflections
*b* = 9.347 (5) Å	θ = 1.9–33.3°
*c* = 11.078 (5) Å	µ = 0.09 mm^−^^1^
α = 99.213 (5)°	*T* = 293 K
β = 102.740 (5)°	Prism, colourless
γ = 97.767 (5)°	0.22 × 0.20 × 0.16 mm
*V* = 655.6 (7) Å^3^	

### Data collection

**Table:**

Bruker APEXII Kappa CCD-detector diffractometer	3169 reflections with *I* > 2σ(*I*)
Radiation source: fine-focus sealed tube	*R*_int_ = 0.036
graphite	θ_max_ = 33.3°, θ_min_ = 1.9°
Detector resolution: 0 pixels mm^-1^	*h* = −10→9
ω and φ scans	*k* = −14→14
18424 measured reflections	*l* = −17→16
4996 independent reflections	

### Refinement

**Table:**

Refinement on *F*^2^	Primary atom site location: structure-invariant direct methods
Least-squares matrix: full	Secondary atom site location: difference Fourier map
*R*[*F*^2^ > 2σ(*F*^2^)] = 0.057	Hydrogen site location: inferred from neighbouring sites
*wR*(*F*^2^) = 0.173	H-atom parameters constrained
*S* = 0.99	*w* = 1/[σ^2^(*F*_o_^2^) + (0.0847*P*)^2^ + 0.1309*P*] where *P* = (*F*_o_^2^ + 2*F*_c_^2^)/3
4996 reflections	(Δ/σ)_max_ = 0.014
183 parameters	Δρ_max_ = 0.30 e Å^−^^3^
0 restraints	Δρ_min_ = −0.29 e Å^−^^3^

### Special details

**Table:**

Geometry. All e.s.d.'s (except the e.s.d. in the dihedral angle between two l.s. planes) are estimated using the full covariance matrix. The cell e.s.d.'s are taken into account individually in the estimation of e.s.d.'s in distances, angles and torsion angles; correlations between e.s.d.'s in cell parameters are only used when they are defined by crystal symmetry. An approximate (isotropic) treatment of cell e.s.d.'s is used for estimating e.s.d.'s involving l.s. planes.
Refinement. Refinement of *F*^2^ against ALL reflections. The weighted *R*-factor *wR* and goodness of fit *S* are based on *F*^2^, conventional *R*-factors *R* are based on *F*, with *F* set to zero for negative *F*^2^. The threshold expression of *F*^2^ > σ(*F*^2^) is used only for calculating *R*-factors(gt) *etc*. and is not relevant to the choice of reflections for refinement. *R*-factors based on *F*^2^ are statistically about twice as large as those based on *F*, and *R*- factors based on ALL data will be even larger.

### Fractional atomic coordinates and isotropic or equivalent isotropic displacement parameters (Å^2^)

**Table:**

	*x*	*y*	*z*	*U*_iso_*/*U*_eq_	
O1	0.22065 (13)	0.57205 (11)	0.58851 (9)	0.0380 (2)	
C2	0.52515 (18)	0.74174 (14)	0.57658 (11)	0.0306 (3)	
N1	0.23543 (18)	0.67259 (14)	0.41251 (11)	0.0406 (3)	
C6	0.64969 (18)	0.74989 (14)	0.70869 (12)	0.0304 (2)	
C5	0.52503 (19)	0.63581 (14)	0.76101 (12)	0.0315 (3)	
C7	0.6205 (2)	0.60943 (15)	0.88018 (13)	0.0377 (3)	
C4	0.32984 (19)	0.55767 (14)	0.70373 (12)	0.0315 (3)	
C3	0.32497 (19)	0.66098 (15)	0.52808 (12)	0.0320 (3)	
C8	0.6664 (2)	0.90474 (14)	0.78630 (12)	0.0332 (3)	
N3	0.21879 (18)	0.45814 (14)	0.74817 (12)	0.0439 (3)	
H3A	0.0968	0.4142	0.7044	0.053*	
H3B	0.2692	0.4379	0.8206	0.053*	
N2	0.38757 (19)	0.76374 (15)	0.38430 (11)	0.0432 (3)	
H2	0.3736	0.7912	0.3129	0.052*	
N4	0.7004 (2)	0.59058 (17)	0.97741 (14)	0.0571 (4)	
C1	0.5621 (2)	0.80702 (16)	0.47848 (13)	0.0376 (3)	
C14	0.8640 (2)	0.70915 (18)	0.70847 (16)	0.0438 (3)	
H14A	0.8467	0.6116	0.6599	0.066*	
H14B	0.9397	0.7124	0.7936	0.066*	
H14C	0.9401	0.7779	0.6718	0.066*	
C13	0.4882 (2)	0.95084 (17)	0.81184 (15)	0.0442 (3)	
H13	0.3617	0.8859	0.7841	0.053*	
C9	0.8513 (3)	1.00406 (18)	0.82993 (17)	0.0517 (4)	
H9	0.9738	0.9768	0.8150	0.062*	
C10	0.8558 (3)	1.1452 (2)	0.89631 (18)	0.0639 (5)	
H10	0.9817	1.2109	0.9251	0.077*	
C15	0.7477 (3)	0.9053 (2)	0.46603 (16)	0.0536 (4)	
H15A	0.8448	0.8469	0.4411	0.080*	
H15B	0.8130	0.9701	0.5456	0.080*	
H15C	0.7051	0.9625	0.4033	0.080*	
C12	0.4943 (3)	1.0905 (2)	0.87732 (17)	0.0568 (4)	
H12	0.3727	1.1186	0.8930	0.068*	
C11	0.6797 (4)	1.18818 (19)	0.91953 (16)	0.0595 (5)	
H11	0.6843	1.2826	0.9635	0.071*	

### Atomic displacement parameters (Å^2^)

**Table:**

	*U*^11^	*U*^22^	*U*^33^	*U*^12^	*U*^13^	*U*^23^
O1	0.0256 (4)	0.0500 (6)	0.0318 (5)	−0.0082 (4)	−0.0019 (3)	0.0142 (4)
C2	0.0260 (5)	0.0314 (6)	0.0302 (6)	−0.0015 (4)	0.0032 (4)	0.0049 (4)
N1	0.0346 (6)	0.0480 (7)	0.0319 (6)	−0.0072 (5)	−0.0007 (4)	0.0116 (5)
C6	0.0233 (5)	0.0309 (6)	0.0322 (6)	−0.0011 (4)	0.0012 (4)	0.0056 (4)
C5	0.0272 (5)	0.0296 (6)	0.0320 (6)	−0.0019 (4)	−0.0014 (4)	0.0073 (5)
C7	0.0308 (6)	0.0350 (7)	0.0397 (7)	−0.0061 (5)	−0.0026 (5)	0.0109 (5)
C4	0.0266 (5)	0.0338 (6)	0.0303 (6)	0.0004 (4)	0.0013 (4)	0.0072 (5)
C3	0.0273 (5)	0.0362 (6)	0.0290 (6)	−0.0015 (4)	0.0029 (4)	0.0073 (5)
C8	0.0330 (6)	0.0318 (6)	0.0286 (6)	−0.0033 (5)	−0.0005 (5)	0.0066 (5)
N3	0.0331 (6)	0.0509 (7)	0.0396 (6)	−0.0111 (5)	−0.0038 (5)	0.0191 (5)
N2	0.0415 (6)	0.0517 (7)	0.0308 (6)	−0.0082 (5)	0.0030 (5)	0.0144 (5)
N4	0.0472 (7)	0.0635 (9)	0.0477 (8)	−0.0143 (6)	−0.0114 (6)	0.0260 (7)
C1	0.0327 (6)	0.0407 (7)	0.0352 (7)	−0.0031 (5)	0.0057 (5)	0.0075 (5)
C14	0.0271 (6)	0.0477 (8)	0.0529 (9)	0.0045 (5)	0.0052 (6)	0.0076 (7)
C13	0.0464 (8)	0.0385 (8)	0.0450 (8)	0.0025 (6)	0.0122 (6)	0.0034 (6)
C9	0.0384 (7)	0.0422 (8)	0.0600 (10)	−0.0085 (6)	−0.0025 (7)	0.0016 (7)
C10	0.0682 (12)	0.0430 (9)	0.0577 (11)	−0.0165 (8)	−0.0087 (9)	−0.0007 (8)
C15	0.0450 (8)	0.0609 (10)	0.0498 (9)	−0.0142 (7)	0.0090 (7)	0.0193 (8)
C12	0.0746 (12)	0.0457 (9)	0.0514 (10)	0.0136 (8)	0.0213 (9)	0.0032 (7)
C11	0.0922 (14)	0.0371 (8)	0.0405 (8)	0.0039 (9)	0.0083 (9)	−0.0001 (6)

### Geometric parameters (Å, °)

**Table:**

O1—C4	1.3602 (16)	N2—C1	1.3465 (19)
O1—C3	1.3611 (16)	N2—H2	0.8600
C2—C1	1.3799 (19)	C1—C15	1.486 (2)
C2—C3	1.3885 (18)	C14—H14A	0.9600
C2—C6	1.5001 (18)	C14—H14B	0.9600
N1—C3	1.3164 (17)	C14—H14C	0.9600
N1—N2	1.3595 (17)	C13—C12	1.379 (2)
C6—C5	1.5289 (18)	C13—H13	0.9300
C6—C14	1.531 (2)	C9—C10	1.397 (3)
C6—C8	1.5369 (19)	C9—H9	0.9300
C5—C4	1.3661 (18)	C10—C11	1.357 (3)
C5—C7	1.4091 (19)	C10—H10	0.9300
C7—N4	1.1440 (19)	C15—H15A	0.9600
C4—N3	1.3340 (17)	C15—H15B	0.9600
C8—C9	1.380 (2)	C15—H15C	0.9600
C8—C13	1.390 (2)	C12—C11	1.374 (3)
N3—H3A	0.8600	C12—H12	0.9300
N3—H3B	0.8600	C11—H11	0.9300
			
C4—O1—C3	115.59 (10)	N2—C1—C2	106.17 (12)
C1—C2—C3	103.33 (11)	N2—C1—C15	122.22 (14)
C1—C2—C6	133.30 (11)	C2—C1—C15	131.61 (13)
C3—C2—C6	123.33 (11)	C6—C14—H14A	109.5
C3—N1—N2	101.74 (11)	C6—C14—H14B	109.5
C2—C6—C5	105.36 (10)	H14A—C14—H14B	109.5
C2—C6—C14	110.78 (11)	C6—C14—H14C	109.5
C5—C6—C14	109.28 (12)	H14A—C14—H14C	109.5
C2—C6—C8	109.09 (10)	H14B—C14—H14C	109.5
C5—C6—C8	109.98 (11)	C12—C13—C8	121.64 (15)
C14—C6—C8	112.12 (10)	C12—C13—H13	119.2
C4—C5—C7	116.92 (12)	C8—C13—H13	119.2
C4—C5—C6	126.31 (11)	C8—C9—C10	120.50 (17)
C7—C5—C6	116.77 (11)	C8—C9—H9	119.8
N4—C7—C5	178.69 (15)	C10—C9—H9	119.8
N3—C4—O1	110.00 (11)	C11—C10—C9	121.11 (17)
N3—C4—C5	127.07 (12)	C11—C10—H10	119.4
O1—C4—C5	122.93 (12)	C9—C10—H10	119.4
N1—C3—O1	119.33 (11)	C1—C15—H15A	109.5
N1—C3—C2	114.94 (12)	C1—C15—H15B	109.5
O1—C3—C2	125.71 (12)	H15A—C15—H15B	109.5
C9—C8—C13	117.40 (14)	C1—C15—H15C	109.5
C9—C8—C6	123.01 (13)	H15A—C15—H15C	109.5
C13—C8—C6	119.57 (11)	H15B—C15—H15C	109.5
C4—N3—H3A	120.0	C11—C12—C13	120.16 (18)
C4—N3—H3B	120.0	C11—C12—H12	119.9
H3A—N3—H3B	120.0	C13—C12—H12	119.9
C1—N2—N1	113.80 (12)	C10—C11—C12	119.19 (17)
C1—N2—H2	123.1	C10—C11—H11	120.4
N1—N2—H2	123.1	C12—C11—H11	120.4
			
C1—C2—C6—C5	−173.79 (14)	C6—C2—C3—N1	176.69 (12)
C3—C2—C6—C5	8.91 (17)	C1—C2—C3—O1	177.46 (13)
C1—C2—C6—C14	−55.7 (2)	C6—C2—C3—O1	−4.6 (2)
C3—C2—C6—C14	126.98 (14)	C2—C6—C8—C9	−111.80 (15)
C1—C2—C6—C8	68.17 (18)	C5—C6—C8—C9	133.10 (14)
C3—C2—C6—C8	−109.13 (14)	C14—C6—C8—C9	11.29 (19)
C2—C6—C5—C4	−7.33 (18)	C2—C6—C8—C13	66.30 (15)
C14—C6—C5—C4	−126.40 (15)	C5—C6—C8—C13	−48.80 (16)
C8—C6—C5—C4	110.12 (15)	C14—C6—C8—C13	−170.61 (13)
C2—C6—C5—C7	173.06 (12)	C3—N1—N2—C1	−0.42 (17)
C14—C6—C5—C7	53.99 (16)	N1—N2—C1—C2	−0.34 (18)
C8—C6—C5—C7	−69.50 (15)	N1—N2—C1—C15	179.23 (15)
C4—C5—C7—N4	−158 (8)	C3—C2—C1—N2	0.91 (15)
C6—C5—C7—N4	21 (8)	C6—C2—C1—N2	−176.77 (14)
C3—O1—C4—N3	−174.36 (12)	C3—C2—C1—C15	−178.61 (17)
C3—O1—C4—C5	5.24 (19)	C6—C2—C1—C15	3.7 (3)
C7—C5—C4—N3	−0.2 (2)	C9—C8—C13—C12	0.5 (2)
C6—C5—C4—N3	−179.80 (13)	C6—C8—C13—C12	−177.74 (14)
C7—C5—C4—O1	−179.72 (12)	C13—C8—C9—C10	−0.4 (2)
C6—C5—C4—O1	0.7 (2)	C6—C8—C9—C10	177.75 (15)
N2—N1—C3—O1	−177.77 (12)	C8—C9—C10—C11	0.0 (3)
N2—N1—C3—C2	1.06 (17)	C8—C13—C12—C11	−0.2 (3)
C4—O1—C3—N1	175.29 (12)	C9—C10—C11—C12	0.3 (3)
C4—O1—C3—C2	−3.4 (2)	C13—C12—C11—C10	−0.2 (3)
C1—C2—C3—N1	−1.29 (17)		

### Hydrogen-bond geometry (Å, °)

**Table:**

*D*—H···*A*	*D*—H	H···*A*	*D*···*A*	*D*—H···*A*
N3—H3A···N1^i^	0.86	2.27	3.129 (2)	173
N3—H3B···N4^ii^	0.86	2.26	3.087 (2)	160
C10—H10···N4^iii^	0.93	2.53	3.455 (3)	172
C14—H14A···N1^iv^	0.96	2.59	3.522 (3)	163

Symmetry codes: (i) −*x*, −*y*+1, −*z*+1; (ii) −*x*+1, −*y*+1, −*z*+2; (iii) −*x*+2, −*y*+2, −*z*+2; (iv) −*x*+1, −*y*+1, −*z*+1.
